# Determinants of tuberculosis: an example of high tuberculosis burden in the Saharia tribe

**DOI:** 10.3389/fpubh.2023.1226980

**Published:** 2023-10-18

**Authors:** Parul Gupta, Pushpendra Singh, Aparup Das, Ravindra Kumar

**Affiliations:** ICMR-National Institute of Research in Tribal Health, Jabalpur, India

**Keywords:** malnutrition, public health, Saharia, tobacco, tuberculosis, catastrophic expenditure

## Abstract

Tuberculosis (TB) is a significant public health problem among the Saharia community, an underprivileged tribal group in the west-central part of India. There are several challenges for India’s TB control program to curtail TB in the Saharia tribe. Malnutrition, poor health sector facilities, lower socio-economic status, and substance abuse are interconnected and synergistic factors contributing to a high burden of TB in the Saharia tribe. In this review, efforts are made to collate the findings of previous studies discussing the causes of high burden of TB in the Saharia tribe, social gaps for mitigating these preventable risk factors of TB in the Saharia tribe, and the plausible solutions for closing these gaps. The concept of *Health in All Policies* and intersectoral co-ordination is needed for the reduction of TB in the Saharia tribe and to make India TB-free by the year 2025.

## 1. Background

Tuberculosis (TB) is an airborne infectious disease, and its persistent morbidity and mortality remains one of the major public health challenges in developing countries such as India (1). Three countries, namely India, Indonesia, and the Philippines, shoulder the 67% global burden of TB (2). It is estimated that, globally, 10.6 million people developed TB in the year 2021 as compared to 10.1 million in the preceding year (3). TB is the 13th leading cause of death and second leading infectious disease globally after coronavirus (4).

India contributes 30% of the global TB cases, which is the highest in the world (2, 5, 6). The incidence of TB in India for the year 2022 was 172 per 100,000 people. A sharp rise in TB cases of 13% was recorded in 2022 as compared with 2021 (6, 7). The prevalence of TB is lowest in Kerala (67 per 100,000) whereas it is highest (546 per 100,000) in the state of Delhi (6–8).

The Saharia community is a particularly vulnerable tribal group (PVTG) (9). The prevalence of pulmonary TB in Saharia PVTG is alarmingly high (about 7–10 times higher than the national average in previous studies). The prevalence of TB in the Saharia tribe has been reported as 1518/100,000 in Sheopur (10), 1504/100,000 in Shivpuri (11), and 3294/100,000 in Gwalior districts (12) in previous surveys conducted at different time points. Various demographic and socio-economic factors as well as nutritional status, availability of health amenities in the vicinity, and lifestyle patterns may be responsible for the high prevalence of TB in Saharia tribe (13).

India is planning to eradicate TB by the year 2025, Five years ahead of the global End TB timeline (1, 14). TB-free initiatives at state levels are being carried out to achieve a 90% reduction in the prevalence of TB by the year 2025 from a baseline prevalence of the year 2015 (3, 15). To attain the goal of END-TB by 2025, it is urgent to mitigate the risk factors associated with TB. In previous studies, various determinants of health associated with the high burden of TB in Saharia PVTG have been identified, which are summarized in this article. In addition, several gaps and challenges for reducing the risk factors in previous studies are also discussed in this article.

## 2. Risk factors associated with the high prevalence of TB among the Saharia tribe

**Figure 1:**
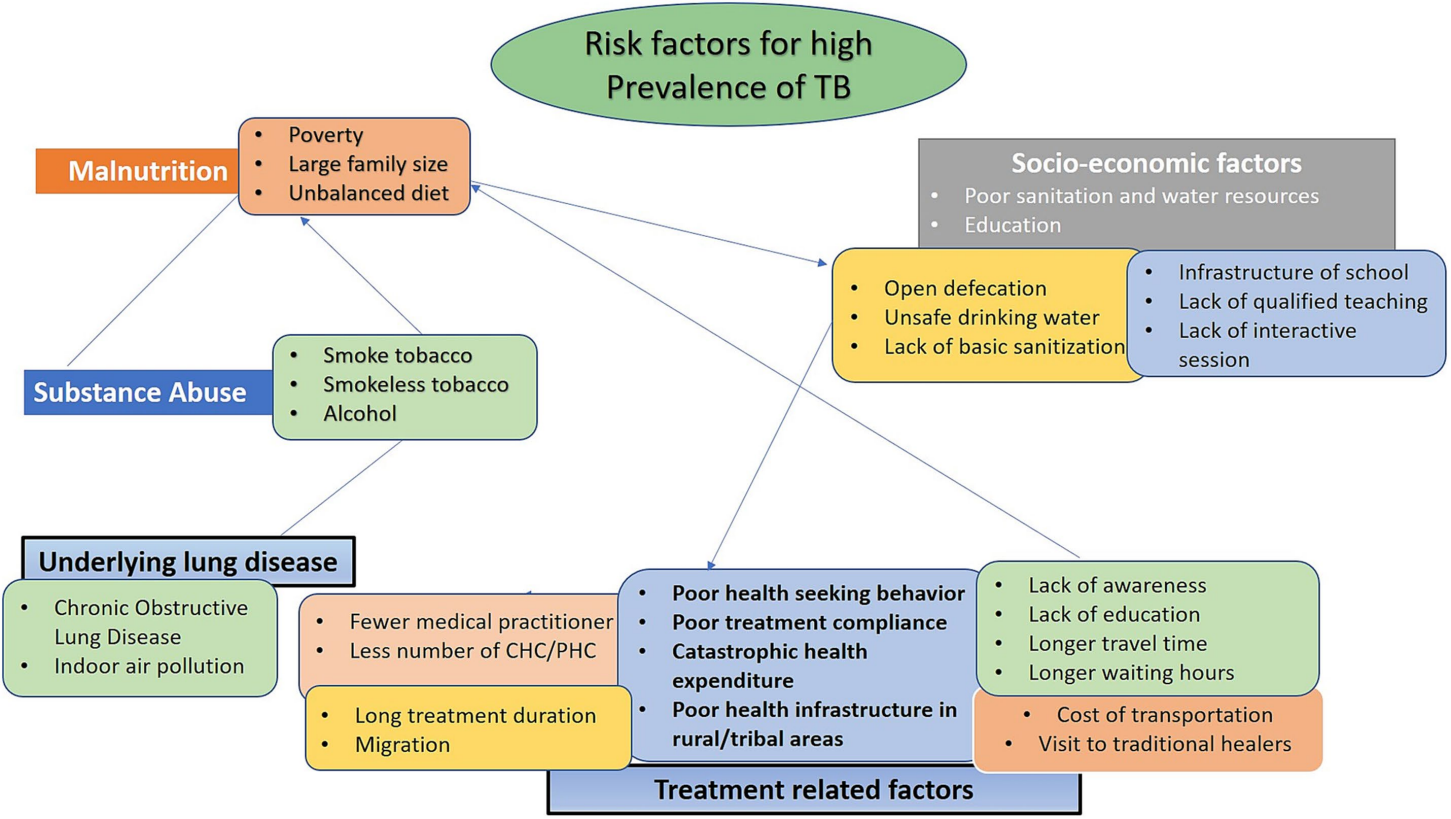
Conceptual framework for interrelation of risk factors responsible for TB.

As discussed above, the Saharia tribe exhibit a high burden of TB. Various factors such as malnutrition, underlying lung disease(s), substance abuse, treatment-related factors, and low-socio-economic status have been found to be linked with TB [(12, 16–18); Figure 1]. These factors need to be addressed in the context of Saharia PVTG.

### 2.1. Malnutrition

**Figure 2:**
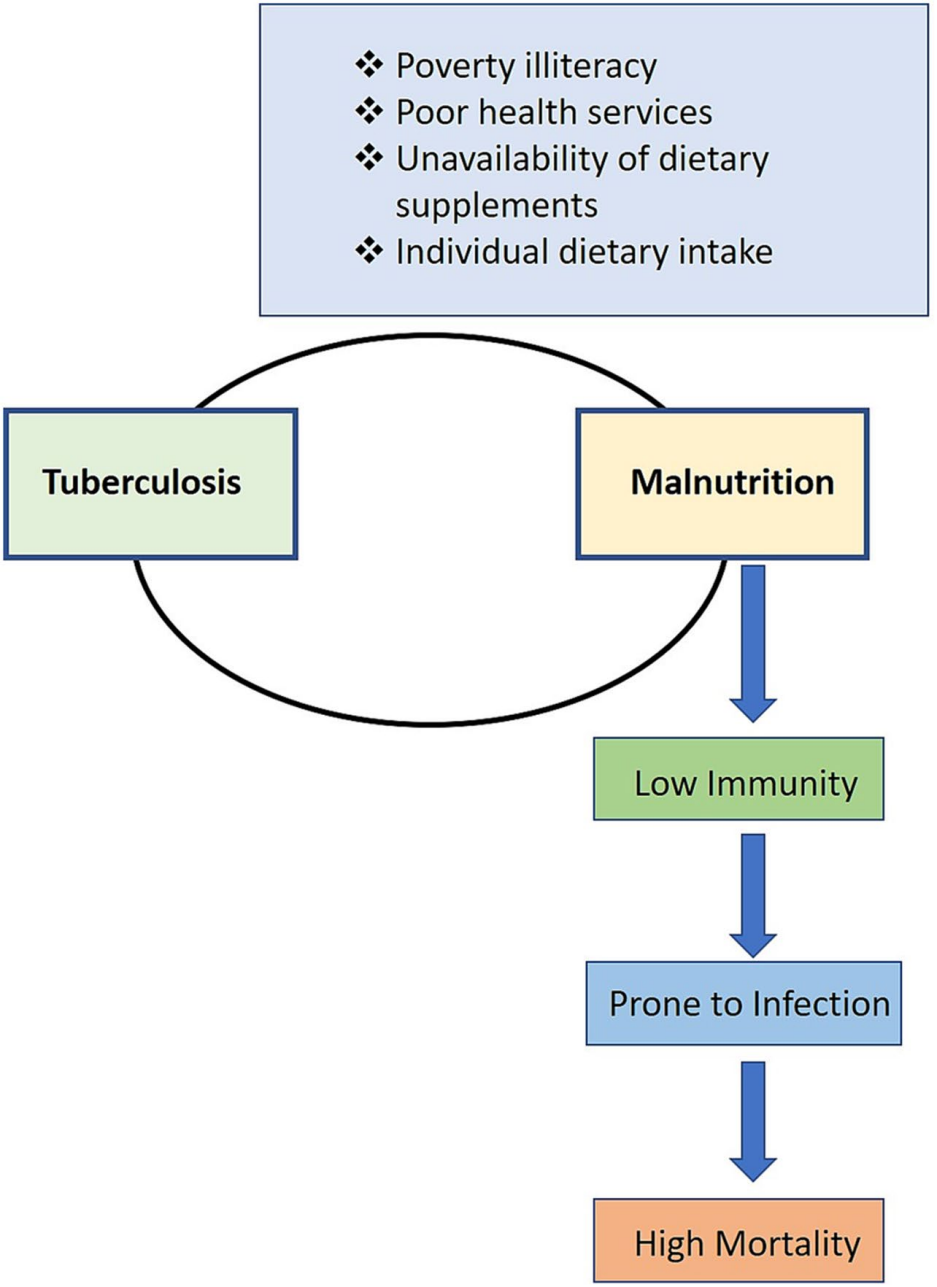
Relationship between TB and malnutrition: Due to TB and malnutrition, the affected person has generally lower immunity and is more prone to infection, which leads to high mortality (19–28).

Undernutrition and TB form a vicious cycle. Malnourished individuals are more susceptible to the development of active TB, while TB exacerbates malnutrition [Figure 2; (19, 20)]. Cell-mediated immunity, which is the principal defense against *Mycobacterium tuberculosis*, is selectively compromised by undernutrition, and thereby increases the risk of reactivation of latent infection to disease (21). Various evidence generated from ecological studies, case series, and large population-based cohort studies have shown the association between nutritional status and the incidence of TB among humans (19, 22, 23). Previous systematic reviews and meta-analysis have shown that the TB incidence reduces by 13.8% with per unit increase in body mass index (BMI) (23, 24). Furthermore, malnutrition also affects the outcome of TB treatment (20). A study conducted in rural Chhattisgarh state, India, by following up 1,179 active TB cases, showed that severe undernutrition at the time of diagnosis was associated with two times higher risk of death (25). There are high chances of relapse of TB in patients who are underweight before the start of treatment (22, 26). Furthermore, TB infection increases the anabolic process and consumes additional energy and therefore TB patients are at increasing risk of becoming underweight (25).

The Saharia tribe have an exorbitantly high rate of malnutrition (12, 27). Previous reports indicate 32–60% of children below 5 years of age are underweight whereas stunting was observed in 40 to 65% of children belonging to Saharia PVTG. Previous studies have also shown that 93% of Saharia children suffered from malnourishment at some age and 15% of them were almost on the verge of death due to malnourishment (28). The high prevalence of undernutrition in the Saharia tribe is also supported by the fact that more than 75.2% non-pregnant non-lactating (NPNL) and 74.6% pregnant women have anemia. Out of these, 4–15% have severe anemia (28). Furthermore, 64% of TB cases were attributed to malnutrition in Saharia PVTG. Additionally, Saharia people with BMI <18.5 kg/m^2^ have a high risk of pulmonary TB [Table 1; (12)].

**Table 1:** Associated risk factors for tuberculosis in Saharia tribe.

Authors’ name	Year	Determinants of TB	Level of association (Odds Ratio, 95%CI)	Value of *p*	Reference
Bhat J. et al.	2017	Malnourished	3.81 (2.61–5.56)	0.001	(12)
Bhat J. et al.	2017	Smoking	1.64 (1.25–2.13)	0.001	(12)
Bhat J. et al.	2017	Tobacco	1.29 (0.99–1.70)	0.059	(12)
Chelleng P. K. et al.	2014	Illiterate	1.65 (1.11–2.46)	0.013	(16)
Rao V. G. et al.	2012	Smoking	1.8 (1.3–2.5)	–	(17)
Rao V. G. et al.	2012	Alcohol	1.7 (1.1–2.6)	–	(17)
Hill et. al.	2006	Family TB history	6.02 (3.23–11.25)	<0.001	(18)
Rao V. G. et al.	2018	Illiterate	0.76 (0.50–1.14)	0.197	(27)
Rao V. G. et al.	2018	Malnourished	1.88 (1.38–2.58)	0.000	(27)
Rao V. G. et al.	2018	Single room	1.18 (0.72–1.94)	0.514	(27)
Rao V. G. et al.	2018	No separate Kitchen	4.11 (1.41–11.99)	0.010	(27)
Rao V. G. et al.	2018	Smoking	1.58 (1.16–2.16)	0.005	(27)
Rao V. G. et al.	2018	Alcohol	1.55 (1.14–2.14)	0.006	(27)
Adilo T. M.	2017	Illiteracy about TB	1.42 (0.88–2.30)	0.001	(29)

### 2.2. Underlying lung diseases and other predisposing factors/conditions

Chronic Obstructive Pulmonary Disease (COPD) is a chronic inflammatory lung disease that causes obstructed airflow from the lungs. The relationship between TB and COPD is complex due to similar symptoms of breathing difficulty, cough, mucus, sputum production, and wheezing. Previous studies showed that patients with chronic COPD have a three times higher risk of developing active TB as compared to the general population (30). The growing global burden of COPD is alarming, especially in highly endemic TB settings (30, 31). Various risk factors such as lower socioeconomic status, air pollution, and exposure to environmental or occupational dust are the major contributing factors for lung diseases like COPD in India (32–34). Exposure to indoor smoke by cooking inside the house using firewood or kerosene is considered the second-most common risk factor associated with COPD (35–38).

**Figure 3:**
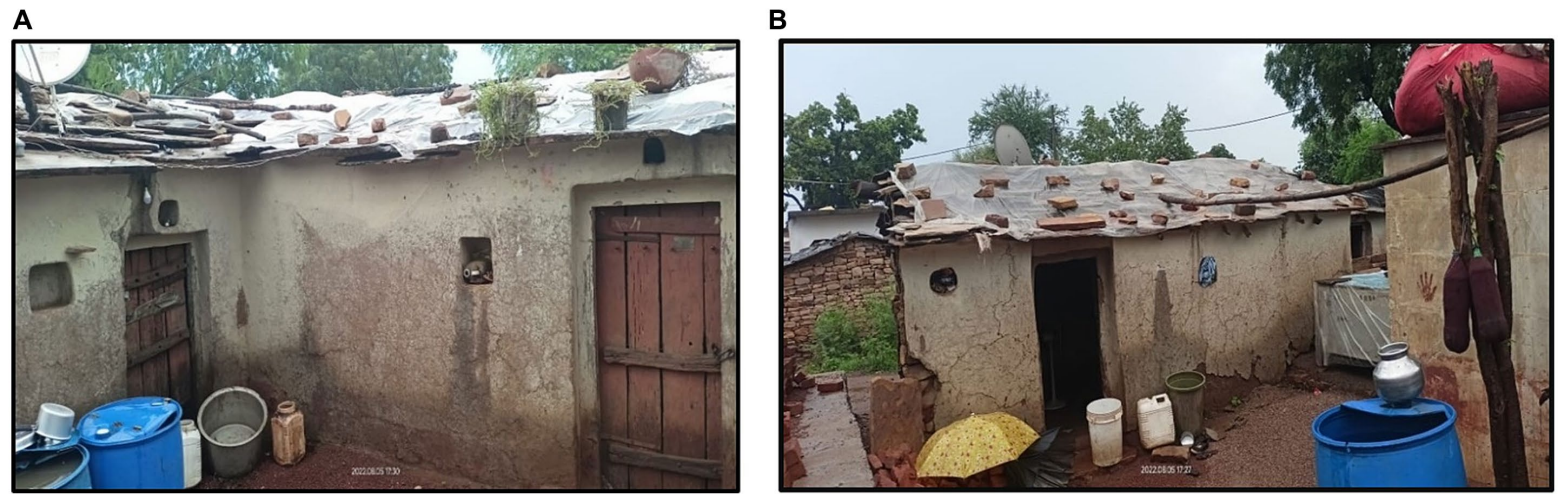
Traditional designs of Saharia houses (**A** and **B**) (lack of ventilation increases the chances of indoor transmission from an index case to other members of the family) (Photos are captured by the authors during field activities).

In the Saharia tribe, 12–18% of people with pulmonary TB also have asthma [Table 1; (16, 28)]. The people of the Saharia tribe usually live in one-room houses with no proper ventilation and cook inside the house using firewood (Figures 3A,B). Therefore, they are exposed to a higher amount of toxic air pollutants and microscopic fine particles produced by biofuels. Frequent exposure to this smoke may lead to burning eyes, bronchitis, COPD, and asthma. Previous studies have shown that cooking inside the house using traditional methods is associated with the occurrence of TB [(27); Table 1].

Furthermore, the majority of the Saharia community works as migratory laborers in rice fields where they are exposed to rice husk dust. Previous studies showed that exposure to rice husk dust is associated with decreased lung function. Long-term exposure may lead to the development of COPD (39–42).

### 2.3. Substance abuse

Substance abuse is a significant risk factor for TB infection and disease (Table 1). Substance abusers present unique challenges for TB diagnosis and control (43). They are more likely to be infectious, take longer to become culture-negative for TB bacilli, and remain at increased risk of TB-related mortality. The physiological effects of substance abuse may contribute to the high prevalence of TB among substance abusers. *In-vitro* studies have shown deleterious effects on the immune system, with biological evidence supporting direct impairment of the cell-mediated immune response using opium-derived drugs (opiates) (44).

Tobacco smoking is a major factor in the loss of lung function and causes lung diseases like TB, COPD, and asthma. Risk of occurring active TB is 1.5 to 2 times higher among smokers (34, 41, 42). Smoking suppresses innate as well as adaptive immunity and reduces the activity of alveolar macrophages, dendritic cells, and natural killer cells. This in turn increases the susceptibility to pulmonary bacterial infection (45). Furthermore, several studies demonstrated alcohol consumption to be a major risk factor for TB (46–49).

Comparative studies have explained that heavy episodic drinking is associated with higher rates of treatment failure, relapse, and death (48). Additionally, alcohol is a factor for default and non-compliance in TB. Moreover, alcohol intake increases the risk of active TB among TB patients. Alcohol and alcohol-related conditions are reported to influence the immune system and contribute to the increase risk of TB in individuals with alcohol use disorders (47).

High rates of tobacco smoking and alcohol consumption in the Saharia tribe may be one of the contributing factors for the high burden of TB. The Saharia tribe have a high rate of alcohol and tobacco consumption which can be exemplified by the fact that around 83% of the Saharia people spend a significant amount of their regular monthly income on alcohol/tobacco products (28). Furthermore, groups of Saharia people often share *bidi* (a cheaper alternative to cigarettes for tobacco smoking, made with *Tendu* leaves locally), which are wet with saliva and thus can contribute to increased transmission of *Mycobacterium tuberculosis* among the social groups (9), considering the already high prevalence of TB in Saharia community members. Previous studies conducted in the Saharia tribe have shown that alcohol consumption and tobacco smoking increase the risk for development of pulmonary TB (27). Studies have shown that the prevalence of respiratory disease remains higher among the members who are involved in the occupation of *bidi* making (50). Other studies revealed that hazardous exposure to tobacco among children is associated with symptoms of ill health, poor nutritional status, and higher risk of infectious diseases, including TB and HIV (51). Despite the source of substitutional income through *bidi* making, it causes hazardous health impacts on the workers, especially children, involved in this trade. During the process of making *bidi*, tobacco powder needs to undergo heat treatment, vaporizing it into air, and the persons working in these areas are inevitably exposed to tobacco smoke through their skin and respiratory tracts. Many members of the Saharia community are engaged in the occupation of ‘*bidi* making’ in their improperly ventilated houses, which makes them even more vulnerable to tobacco exposure (50).

### 2.4. Treatment-related factors

#### 2.4.1. Poor health-seeking behavior

**Figure 4:**
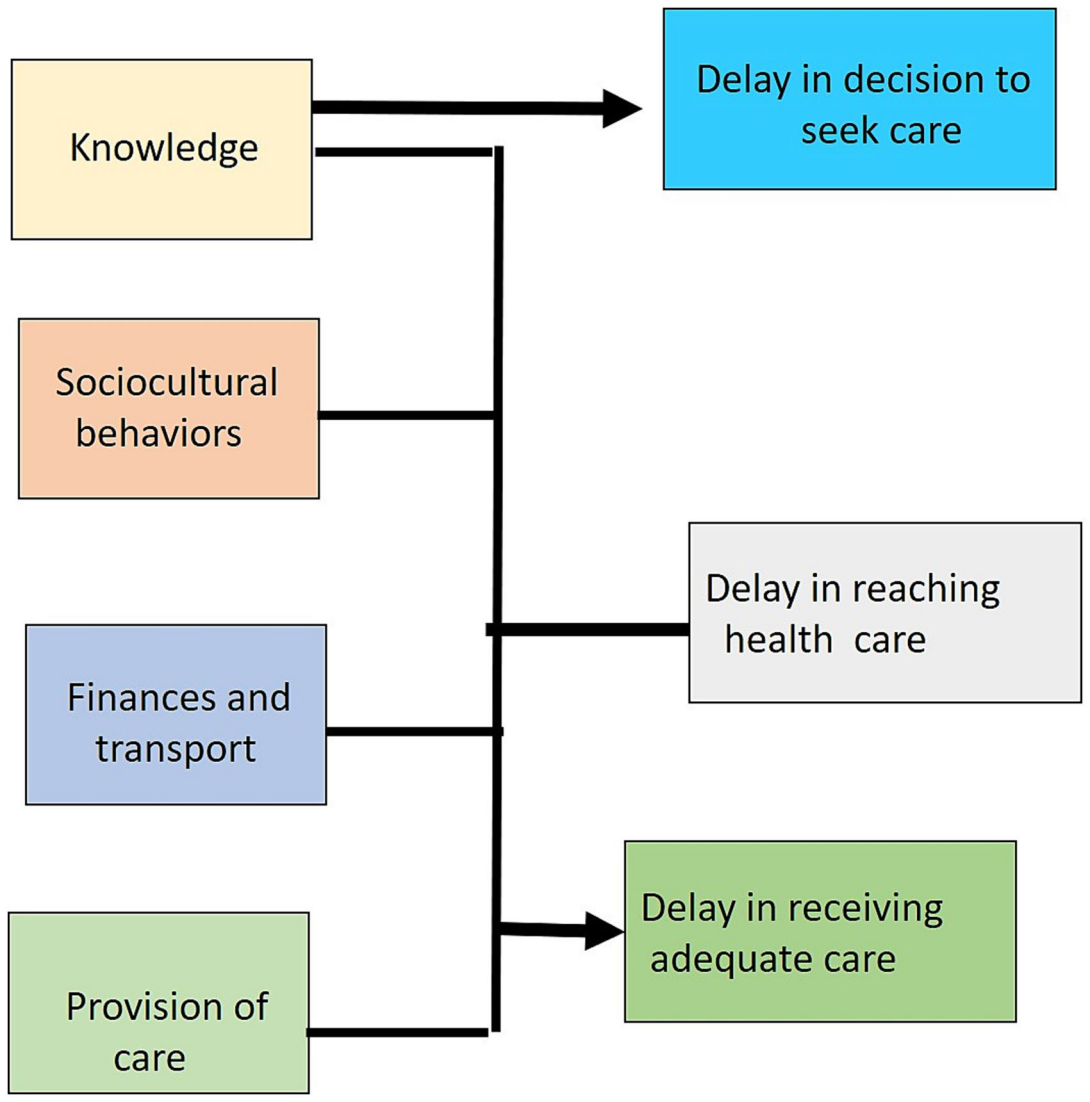
Interconnected factors for poor health-seeking behavior: Various factors like lack of awareness, longer travel time to reach hospitals, and catastrophic expenditure on TB treatment are responsible for poor health-seeking behavior (52–54).

Like other tribes, Saharia also has poor health-seeking behavior due to various factors (Figure 4): (i) socio-cultural beliefs in traditional healers (called ‘*Gunias’),* (ii) lack of awareness and faith in modern treatment facilities, (iii) lack of health facilities in their vicinity, and (iv) lack of public transport and health illiteracy (52, 53). Saharia community members generally use take-home remedies to cure diseases and usually prefer to visit traditional healers. Belief in local practices and *Gunias* indirectly affects health outcomes adversely, thereby further contributing to transmission of TB bacilli [(1, 53); Table 1].

Since TB is an airborne disease, early treatment is crucial for preventing transmission. Various factors are responsible for the delay in diagnosis and treatment of TB patients. Poor health seeking behavior is one of the major risk factors associated with high transmission of TB. The treatment of 30.5% of TB cases is delayed due to lack of awareness. Another major reason for delay in treatment initiation among 43.17% of TB cases is due to their visit to traditional healers/private practitioners after TB diagnosis in tribes of the Odisha state (52). However, similar information in the Saharia tribe living in Madhya Pradesh state is lacking. Various factors like illiteracy, lack of awareness, and treatment expenditure are contributing to poor health-seeking behavior of the Saharia tribe [(52–54); Figure 4].

#### 2.4.2. Poor treatment compliance

Longer treatment duration with various side effects for anti-tubercular therapy (ATT) may be a responsible factor for poor treatment compliance (29, 54, 55). Previous studies reported that 23% of patients left their treatment early because of the long time duration (28). Most of the Saharia are daily wage earners. Among the Saharia households, approximately 56% migrated for work while only 12% of those from non-Saharia households migrated for work (28). Due to this migration, the Saharia tribe has poor treatment compliance and, therefore, increased transmission of *Mycobacterium tuberculosis* (55).

#### 2.4.3. Catastrophic health expenditure

Despite having free diagnosis and treatment of TB from the National Tuberculosis Elimination Program (NTEP), various out-of-pocket expenditures are incurred by the families affected with TB. The Saharia tribe face catastrophic TB care expenditures that can amount to roughly 10% of their annual income (28). Catastrophic cost includes the loss of daily wages and travel costs to healthcare facility. Moreover, treatment and hospitalization in severe cases exert a higher economic burden on TB patients (56). Expenditure caused by TB and its treatment can be catastrophic for households (57).

#### 2.4.4. Poor health infrastructure in rural/tribal areas

Among the barriers that restrict TB control in Saharia is poor healthcare facilities. Nine Saharia-inhabited districts of Madhya Pradesh have a total population of approx. 1.07 crores. These nine districts have only 52 Community Health Centers, which is half of the required CHC as per NHM guidelines (58). Similarly, there are only 25% of the necessary Primary Healthcare Centers (PHCs) compared to the recommended number.

### 2.5. Socio-economic factors

#### 2.5.1. Poor housing and overcrowding

The End TB Strategy and UN Sustainable Development Goals recognize the interdependence between social determinants and health, particularly in low-and middle-income countries where most TB cases are clustered among economically and socially disadvantaged groups. Furthermore, previous studies carried out in the Saharia community have reported that poor housing quality and overcrowding correlate with increased TB prevalence, while higher ‘social capital’ (detailed below) was associated with lower TB prevalence within a household (59). ‘Social capital’ refers to the value of social networks and connections, which may promote dissemination of TB-related awareness, leading to increase knowledge and TB-preventive behavior. Furthermore, overcrowding and roof leakage significantly contributed to the increased probability of TB (59, 60). Due to limited resources, Saharia PVTG are living in small, non-ventilated houses with a large number of family members, making them more prone to TB infection. These findings suggest that socioeconomic factors play a significant role in the high prevalence of TB in the Saharia community. Socioeconomic status is a crucial determinant of differential exposure, vulnerability to disease-causing agents, and differential consequences of ill health.

#### 2.5.2. Poor sanitation and water resources

The Saharia are a vulnerable population with limited access to health resources and services, and they are particularly at risk of TB due to poor sanitation and water resources. Sanitation and water resources are essential for preventing and treating TB, and the lack of access to these resources is a major contributing factor to the high prevalence of TB among the Saharia. Improving access to sanitation and water resources is essential for reducing the prevalence of TB among this population. The unhygienic living conditions increase the likelihood of contracting and spreading the disease, as well as other infectious diseases. Studies have shown the high prevalence of TB in areas with poor sanitation (61). Saharia-inhabited areas of Madhya Pradesh have 35% individual household latrine (IHHL) coverage (62) and most of them continue to defecate in the open (63).

#### 2.5.3. Education

TB is associated with low education levels and reflects the complex interaction between non-communicable disease, urbanization, and a changing economic climate (48, 62). Low education or illiteracy has been found to be associated with TB among the Saharia tribe (64, 65).

Despite the occurrence of many government and private schools in Saharia-inhabited districts of Madhya Pradesh, approximately 40% of the population is still illiterate. The Saharia community has 11% more illiterate people than the non-Saharia community (28).

## 3. Strategies for reduction of preventable risk factors associated with TB

As discussed earlier, there are several risk factors (Box 1) which may be attributed to the high prevalence of TB in the Saharia tribe. To eliminate TB in high endemic settings, these risk factors need be addressed. Most of these factors are associated with initiatives for mitigating the above-mentioned risk factors and are discussed below.

**BOX 1:** Highlights on risk factors which may responsible for the prevalence of tuberculosis.

**Malnutrition** 55% of children below 5 years of age in the Sheopur District were underweight. Various National and State level schemes are running to tackle malnutrition in children and women. No scheme to cure malnutrition in adult males. **Underlying Lung disease** Approx. 3 times increased risk of COPD with prior pulmonary TB Smoke-causing traditional cooking was considered as the second most associated risk factor for COPD **Substance abuse** Risk of occurring active TB is 1.5–2.0 times higher among smokers. 83% of Saharia residents spend a part of their monthly income to purchase tobacco and related products. **Poor health seeking behavior** Treatment of 30.5% cases were delayed due to lack of awareness. 43.17% of treatment delay was observed due to visits to traditional healers. **Poor treatment compliance** 23% of Saharias left their treatment due to the long treatment course. 56% Saharias migrated for work. **Catastrophic health Expenditure** The Saharia tribe spends 10% of their annual income on catastrophic TB care expenditure. **Poor Health Infrastructure** 133 PHC out of 500 52 CHC out of 100 CHC 1593 subcenters out of 2500 Still there is a gap between the facilities required and the facilities available. **Poor sanitation and hygiene** Saharia-dominated areas of MP have 31% IHHL coverage. **Education** 40% of Saharias are illiterate.

To overcome malnutrition in tribes, various national-level programs are continuously running such as the “*Pradhan Mantri Matru Vandana Yojana”* (66) and the Scheme for Adolescent Girls under the umbrella of the Integrated Child Development Services Scheme (ICDS) (67). Recently, the POSHAN 2.0 *Abhiyaan* (Prime Minister’s Overarching Scheme for Holistic Nutrition) has also been initiated to curb malnutrition and improve nutritional outcomes for children, pregnant women, and lactating mothers.

Madhya Pradesh is the first state in the country where the community-based Nutrition Management (C-SAM) (66–69) was launched in the form of a two-phase campaign to identify severely malnourished children. Apart from these, various innovative schemes like “*Tiranga Thali*,” “*Dastak Programme*,” and “*Seven (7) days Seven (7) plots*” were started to create awareness, and provide a balanced diet to reduce malnutrition by the Madhya Pradesh government (66). In addition, to tackle the problem of malnutrition in TB patients, the Government of India is supporting the nutritional requirement of all TB patients through *Nikshay Poshan Yojna* for the entire duration of TB treatment (69).

**Figure 5:**
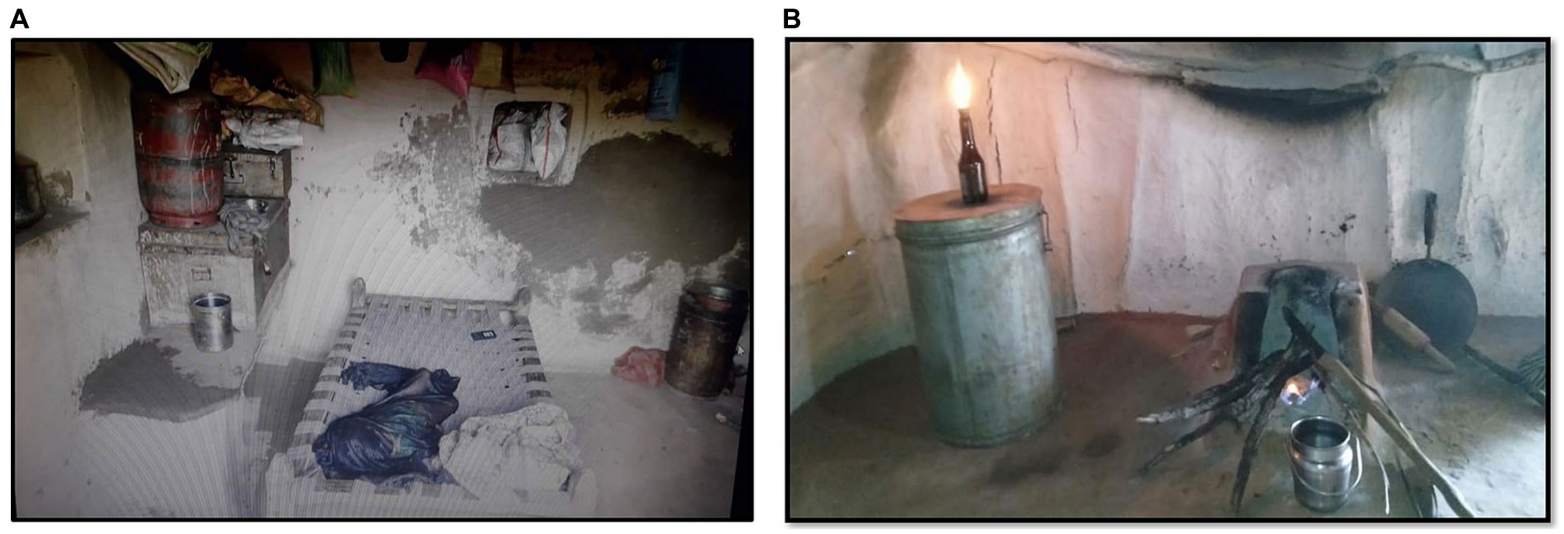
Negligence of LPG cylinders over basic cooking using wood-based smoke *Chulha*: Refilling of gas cylinders is not a routine practice in tribal areas owing to cost constraints **(A)** or distance/supply at home and the easy availability of firewood material from adjoining forests. It encourages more frequent use of smoke fuel and negligible use of LPG **(B)** (Photos are captured by the authors during field activities).

Similarly, to control indoor as well as outdoor environmental pollution and minimize the respiratory health issues that occur due to coal, firewood, and cow dung smoke used for cooking*, Pradhan Mantri Ujjwala Yojana* (70) was launched by the Government of India. *Ujjwala Yojana* provides LPG connections to the remotest and poorest parts of the country to provide a smoke-free cooking environment. However, due to the high cost involved in cooking gas, the majority of Saharia people are still dependent on traditional cooking methods (Figures 5A,B).

Tobacco consumption exerts hazardous direct effect on an individual’s health. At the same time, it also puts creates a high cost burden due to the cost of treatment of tobacco-related diseases. To create awareness about the harmful effects of tobacco consumption and to reduce the production and supply of tobacco products, in the year 2007–2008 the Government of India initiated the National Tobacco Control Programme (NTCP) (71). The program works at the national, state, and district levels.

The Government of India initiated several awareness schemes to prevent irregularity and poor treatment compliance. Migration certificates through the NIKSHAY portal is being provided to migrant people on ATT drug to minimize poor treatment compliance among migratory workers. Despite this, most of the patients become defaulters due to their sudden migration and are lost to follow-up. Regular work opportunity in their areas is the key to reducing migration and related poor treatment compliance.

The loss of daily income and frequently unaffordable expense of treatment push marginalized Saharia TB patients and their families deeper into poverty. For reduction of out-of-pocket expenditure, NTEP initiated doorstep diagnosis and provides treatment through ASHA. Further, NTEP is also providing travel costs for treatment initiation in tribal communities. Policies for reducing catastrophic costs may help in better treatment compliance. In addition to this, the Government of India introduced *Ayushman Bharat*-Health and Wellness Centers (HWC) in Feb 2018, to make the healthcare services available to out-of-reach patients. These HWCs cover around 3,000–5,000 people (72). Integrating TB services with these HWCs decentralizes the healthcare services and increases patient’s footfall for seeking treatment. It reduces the time for start of treatment after diagnosis, improves treatment compliance, and reduces out-of-pocket expenditure.

India has made tremendous progress in ending open defecation which significantly improves Water, Sanitation, and Hygiene (WASH). A tremendous achievement is possible because of the Government’s flagship program, the *Swachh Bharat Mission* (SBM) (Clean India Campaign), with the partnership of UNICEF (73). It has also been observed that there is a reduction in undernutrition in children under five after the increase in household toilet availability (74).

All these policies and programs initiated by the government are not directly benefiting Saharia PVTG. Other programs are indirectly benefiting Saharia such as *Nikshay Poshan Yojna* supporting malnourished TB patients. Similarly, *Ujjwala Yojna* provides gas cylinder connections to enable smoke-free cooking, as cooking-related smoke exposure is a major risk factor of TB. However, studies related to the effect of these initiatives on the reduction of TB in the Saharia tribe are lacking.

## 4. Existing gaps need to be considered

Despite various programs and schemes, Saharias remain among the most marginalized population in Madhya Pradesh and many of these government schemes remain underutilized by the Saharia community. Malnutrition continues to be a persisting challenge and will remain so until the underlying causes of malnutrition in Saharia are resolved. The policymakers and stakeholders consider malnutrition as a health-related issue. However, poverty, unemployment, and lifestyle are also important factors responsible for undernutrition due to an unbalanced diet. Currently, a major focus of various programs is on malnutrition in children and women. Malnutrition among adult males and older people often remains neglected, and these populations are also more susceptible to developing TB (75). Nutrition in adults may be improved through targeted supplementation of micronutrients and protein where diet alone is not sufficient to meet the age-specific requirements (76, 77). Still, there are many challenges for the treatment of malnutrition in adults due to various underlying age-related chronic disease (76).

**Figure 6:**
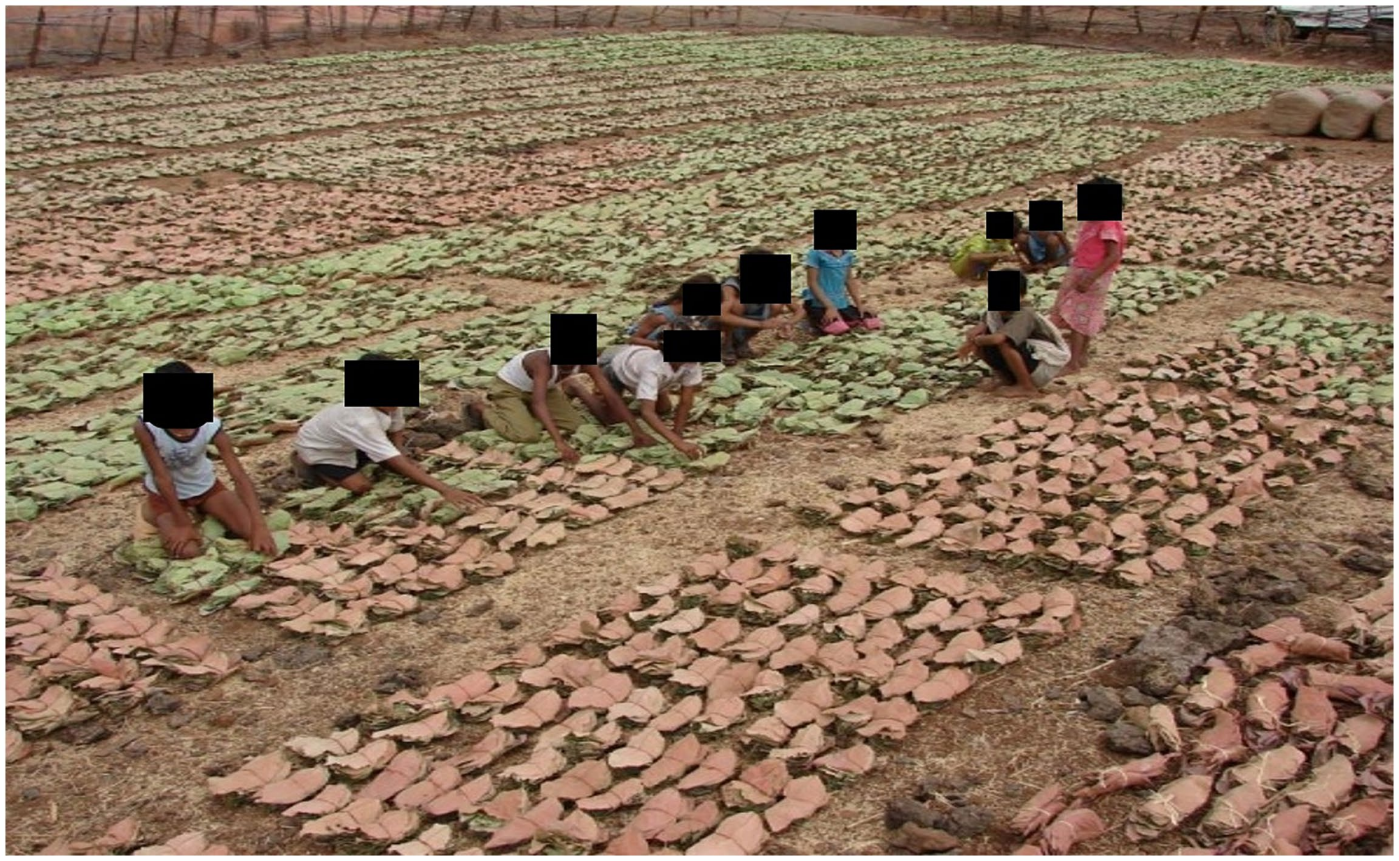
Collection of *Tendu Patta* by Saharia tribe: *Bidi* making process requires filling of tobacco inside *Tendu Patta* leaves which is collected by all age groups including young children. *Bidi* smoking is also a common practice among the workers, especially among adolescents and children from a young age (Photos are captured by the authors during field activities).

Despite awareness about the health hazards of tobacco, reduction in tobacco consumption and substance abuse at the periphery is challenging. Clinicians and public health practitioners should be aware of special considerations and the latest evidence concerning TB management among people who are addicted to substances (43, 44). Strong political will is required to control tobacco consumption which is lacking at present due to the large economical factor involved in the tobacco industry. It is estimated that 20 million people are working in the tobacco industry, in which 0.74 million get employment for about three months through the collection of *tendu* leaves (Figure 6) and 4.16 million people earn their living by making bidis from *Tendu* leaves (78). Of these 4.16 million people, nearly 90% are women and children as this group often works for lower wages and yet remains proficient in rolling bidis (78, 79), thereby increasing the profit margin of the bidi manufacturers.

Timely and accurate diagnosis is the first and foremost requirement for the eradication of TB. Access to medical services should be provided to ensure that every case of TB can be properly diagnosed and treated. Without this assistance, the Saharia tribe will continue to face a high risk of developing TB and associated morbidity and mortality. Additionally, treatment strategies should incorporate the use of directly observed therapy (DOT) with deaddiction program for substance abuse (43, 44). Despite higher expenditure on the treatment of tobacco-related diseases (Rs 177,340 crore) than the average annual revenue collection from tobacco products (Rs 53,750 crore), the reduction of tobacco consumption seems far-sighted (78, 79). Commercial tobacco control strategies like increasing tobacco prices and anti-tobacco mass media campaigns can be effective in reducing the consumption of tobacco products. Deaddiction programs at workplaces may prevent direct as well as involuntary exposure to smoke. There is a high priority to focus on deaddiction and individual-level counseling in tribal areas.

To resolve the problem of open defecation, several toilets have been constructed in tribal and rural areas under the ‘*Swachh Bharat Mission’* (SBM). However, most of the toilets constructed under this mission are being used as store rooms or to wash utensils by the Saharia tribe and they continue to defecate in the open. There is a need to adopt Behavioral Change Communication (BCC) strategies for the effective utilization of toilets and to stop open defecation.

To reduce the illiteracy rate, the education awareness strategy should be revised. There should be a separate strategy for each community. Campaigns for education through interactive meetings with the target community giving examples of successful educated persons belonging to similar communities may be an effective strategy for improving the literacy rate. A small contribution from every educated person to creating awareness toward education may be key to increasing awareness and literacy. Regular monitoring activities by the higher authorities in the schools situated in remote areas may increase attendance at school and improve the quality. Apart from the awareness, adapting the curriculum to suit specific areas may help in the reduction of preventable diseases such as TB. Since TB is a major problem of the Saharia tribe, awareness generation through educational programs at the school level will help in the elimination of TB.

## 5. Conclusion and the way forward

Morbidity and mortality due to TB are unacceptable considering the availability of highly effective early diagnostic and therapeutic options. Various aspects among particularly vulnerable tribal groups such as malnutrition, poor socio-economic status, high rate of substance abuse, and unequal access to diagnosis and treatment pose obstacles to TB elimination programs. Additionally, the increase in COVID-19 combined with the intermittence of health services during the pandemic has led to a predicted increase in the TB burden worldwide (80). To achieve the END TB goal by 2025, there is an urgent need to mitigate these obstacles along with active case finding and prompt treatment in vulnerable tribal groups, which have a high prevalence of TB at present. A context-specific, tailor-made policy and implantation plan based on existing and new scientific evidence is the need of the hour for the elimination of TB in the Saharia tribe. Intersectoral coordination is needed with the concept of *Health in All Policies* for making India TB-free. All concerned ministries, i.e., the Ministry of Health and Family Welfare, Ministry of Tribal Affairs, Ministry of Agriculture, Ministry of Women and Child Development, Ministry of Education, Ministry of Labour and Employment, and the Ministry of Social Justice and Empowerment may come forward and work together to mitigate all possible risk factors associated with TB and eventually eliminate TB from India.

## Author contributions

PG did the literature search and wrote the first draft of the manuscript. RK and AD designed the study and revised the manuscript. PS provided important intellectual suggestions for improving the manuscript. All authors contributed to the article and approved the submitted version.
